# An updated systematic review of pharmacological treatments for presbyopia

**DOI:** 10.1016/j.aopr.2024.09.001

**Published:** 2024-09-03

**Authors:** Andrzej Grzybowski, Laura Kapitanovaite, Reda Zemaitiene

**Affiliations:** a Institute for Research in Ophthalmology, Poznan, Poland; b Department of Ophthalmology, University of Warmia and Mazury, Olsztyn, Poland; c Department of Ophthalmology, Hospital of Lithuanian University of Health Sciences, Kaunas Clinics, Kaunas, Lithuania; d Department of Ophthalmology, Medical Academy, Lithuanian University of Health Sciences, Kaunas, Lithuania

**Keywords:** Presbyopia, Presbyopia treatment, Medical, Pharmacological presbyopia treatment

## Abstract

**Background:**

Presbyopia, a common age-related condition affecting near vision, impacts over a billion people worldwide. The aim of this paper is to review the main reports and results of clinical trials, comparing the newest pharmacological treatment options for presbyopia, their mechanisms of action, and possible side effects.

**Main text:**

Pharmacological approaches, involving eye drops that target the underlying mechanisms of presbyopia, have gained growing interest. Two key pharmacological agents in this field are miotics and lens softeners. Miotics enhance near vision temporarily by creating a pinhole effect, though they may cause side effects and are under further investigation for long-term use with ongoing research also exploring the potential benefits of combining them with other drugs to improve outcomes and reduce adverse reactions. Lens softeners, on the other hand, aim to restore the flexibility of the lens, addressing one of the primary causes of presbyopia. Despite early trials, further development of lens softeners has been suspended. A notable advancement in this field is the recent FDA approval of 1.25% and 0.4% pilocarpine, a miotic agent, for presbyopia treatment. This milestone highlights the growing interest in pharmacological solutions for presbyopia, with several new drugs and their combinations currently being investigated for potential FDA approval.

**Conclusions:**

pharmacological treatments, particularly miotics like pilocarpine, represent a promising alternative to conventional methods for managing presbyopia. Continued research into these treatments, especially combinations of drugs, may offer more effective and convenient options for presbyopia patients in the future.

## 1 Introduction

Presbyopia is a refractive condition that develops with aging, leading to the inability of the eye to accommodate and focus properly on objects at different distances, starting with near vision.^1^ In developing world populations, the lack of awareness and affordability for treatment has led to unmanaged presbyopia affecting up to 50% of individuals over 50 years old. In developed countries, the prevalence is also significant, reaching up to 34%.^1^ Although the inability to focus on objects appears suddenly, loss of accommodation is a slow and progressive process that begins in childhood.^2^ Accommodation, the mechanism enabling the eye to adjust focus, involves ciliary muscle contraction, pupillary constriction, and convergence. The primary cause of presbyopia is lens stiffening, aging of the ciliary muscle and the zonules and changes in biomechanical properties of the parts involved in the accommodation.^3^ Current treatment options include optical correction with spectacles or contact lenses and surgical procedures like corneal or intraocular surgeries. Each of these methods are popular amoung patients, but they do not fully restore eye's accommodation, and they have some drawbacks such as exacerbation of dry eye symptoms, inconvenience, reduced stereopsis, or surgical risks.^4^ Pharmacological approaches have gained attention, with the recent two U.S. Food and Drug Administration (FDA) approvals of pilocarpine hydrochloride eye drops marking a milestone in presbyopia treatment.^5^^,^^6^ Pharmacological approach provides a local, non-invasive, and reversible option for presbyopia treatment. Main pharmacological approaches are classified into two groups: agents that aim to induce miosis while increasing depth of focus though pinhole effect and agents that preserve soft lens.^3^ This article is a continuation of a review published by the authors in 2022.^7^ The aim of this paper is to review the main reports and results of clinical trials and compare newest pharmacological treatment options for presbyopia, their mechanisms of action and possible side effects.

## 2 Methodology

**Fig. 1:**
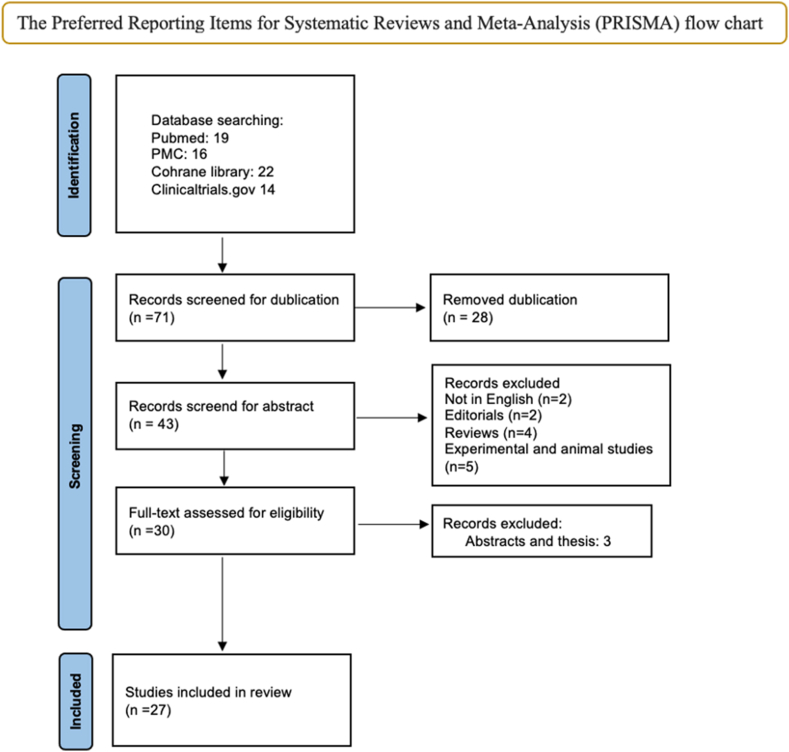
PRISMA flow chart

Our methodology follows the Preferred Reporting Items for Systematic Reviews and Meta-Analysis (PRISMA) guidelines.^8^ The papers were identified by a literature search of medical and other databases (PubMed/MEDLINE, PubMed Central (PMC), Cochrane Library) between November2021 and February 2024. It is a continuation of previously published review article which analysed articles from 2010 to November 2021.^7^^,^^9^ (Fig. 1).

Two trained authors conducted the search strategies and independently screened the titles and abstracts. They selected relevant studies based on the following terms: (presbyopia) AND (medical) AND ((treatment) OR (management) OR (therapy)), (presbyopia) AND (pharmacological) AND ((treatment) OR (management) OR (therapy)). They also searched for undergoing research listed in clinicaltrials.gov by using the following terms: presbyopia, medication, pharmacology. Duplicates were removed at the screening. The preliminary search was based on abstracts. Only peer-reviewed articles in English were considered. Studies presented as editorials, reviews and experimental studies were excluded. In case of discrepancies, they reviewed the article together and reached a consensus, providing reasons for their decision. After the preliminary manual search, 13 papers and 14 trials were selected for further analysis.

## 3 Results

To this day, there are two proposed mechanisms of action for pharmacological treatment of presbyopia including miosis and softening of the lens. There are no new promising developments with the lens softeners since the last review.^7^ Results from clinical trials of the lens softener UNR844, previously known as EV06 (1.5% lipoic acid choline ester), initially showed promise.^10^ However, the pharmaceutical company Novartis has ceased its development due to the drug not achieving a statistically significant dose response in a phase 2b trial at the 3-month mark.^11^ Another drug – STN1013600 ophthalmic solution (ursodeoxycholic acid) was evaluated for the effectiveness and safety in phase IIa clinical trial (NCT05665387) in individuals with presbyopia.^12^ Trial suggests that ursodeoxycholic acid could improve the elasticity of the crystalline lens, however pharmaceutical company Santen has discontinued development of the drug in 2024 following the review of trial data.^13^

The ongoing trials on miotic agents are summarized in Table 1.

**Table 1:** Studies on pharmacological treatment for presbyopia from clinicaltrials.gov.

Drug	N	Study Design	Instillation Method	Primary Outcome Measures	NCT Number	Phases
1.25% pilocarpine^14^	45	Non-randomized, parallel, single group study	Single drop unilaterally	Change of near vision after 1 ​h	NCT05564832	1
0.75% Phentolamine (Nyxol)^15^	333	Multi-center, randomized, double-masked, placebo-controlled study	Single drop binocularly	Percent of subjects with ≥15 letters gain in photopic binocular DCNVA at 30 ​min	NCT05646719	3
Brimochol (Carbachol and Brimonidine tartrate)^16^	182	Multi-center, randomized, double-masked, crossover study	Single drop binocularly	Change in NVA, without the loss of ≥ 1-line in DVA, from baseline to 6 ​h	NCT05270863	3
1% pilocarpine^17^	25	Single group study	Single drop binocularly	Comparison of the BCNVA before and after 20 ​min	NCT05578001	3
LNZ101 (Aceclidine/Brimonidine) and LNZ100 (Aceclidine)^18^	62	Multi-center, randomized, triple-masked, crossover study	Single drop binocularly	Percentage of subjects with ≥3-line improvement in NVA after 1 ​h	NCT05294328	2
LNZ101 (Aceclidine/Brimonidine)^19^	229	Multi-center, randomized, quadruple-masked, parallel-groups study	Single drop unilaterally	Percentage of subjects with ≥ 3-line improvement compared with vehicle, from baseline to 3 ​h post-treatment	NCT05728944	3
LNZ101 (Aceclidine/Brimonidine) and LNZ100 (Aceclidine)^20^	300	Multi-center, randomized, double-masked, placebo-controlled, parallel-groups study	Single drop unilaterally	Percentage of subjects with ≥ 3-line of monocular BCDV at 40 ​cm and no loss in BCDVA of ≥5 letters, from baseline to 3 ​h post-treatment	NCT06045299	3
LNZ101 (Aceclidine/Brimonidine)^21^	361	Multi-center, randomized, triple-masked, parallel-groups study	Once daily binocularly for 28 weeks	Percentage of subjects with AE and monocular BCDVA changes during 7 visits over 28 weeks	NCT05753189	3
LNZ101 (Aceclidine/Brimonidine)^22^	469	Multi-center, randomized, quadruple-masked, parallel-groups study	Single drop binocularly	Percentage of subjects with ≥ 3-line gain with no loss in BCDVA ≥5 letters, from baseline to 3 ​h post-treatment	NCT05656027	3
LNZ100 (Aceclidine) and LNZ101 (Aceclidine/Brimonidine)^23^	58	Multi-center, randomized, triple-masked, crossover study	Single drop binocularly	Percentage of subjects with ≥ 3-line improvement in NVA, 1 ​h post treatment	NCT05431543	2
LNZ100 (Aceclidine) and LNZ101 (Aceclidine/Brimonidine)^24^	30	Single-center, open-label, randomized, parallel-groups study	Once daily binocularly for 8 days	Cmax, Tmax, AUC0-t, AUC0-∞, T1/2 ​at day 1 and 8	NCT05936489	1
LNZ100 (Aceclidine) and LNZ101 (Aceclidine/Brimonidine)^25^	21	Single-center, randomized, double-blind, placebo-controlled, multiple-dose study	Once daily with 1 drop binocularly followed by another drop 2 ​min later for 7 days	Cmax, Tmax, AUC0-t, AUC0-∞, T1/2 ​at day 1 and 8, safety and AE up to 23 days post- treatment	NCT06270030	1
CSF-1 (0.4% Pilocarpine)^6^	178	Multi-center, double-masked, vehicle-controlled study	One drop bilaterally twice daily for 6 weeks	Number of subjects with treatment emergent AE at 6 weeks	NCT05393895	3

Abbreviations: NVA – near visual acuity, BCDVA – best distance corrected visual acuity, DCNVA – distance corrected near visual acuity, DVA – distance visual acuity, BCNVA – best corrected near visual acuity, LNZ101 – Aceclidine/Brimonidine, LNZ100 - Aceclidine, Cmax - maximum plasma concentration, Tmax – time of maximum plasma concentration, AUC0-t – area under the concentration-time curve from time 0 to time t, AUC0-∞ – area under the concentration-time curve from time 0 to infinity, T1/2 – terminal half-life, AE – adverse events.

## 4 Discussion

### 4.1 FDA approved agents

**Fig. 2:**
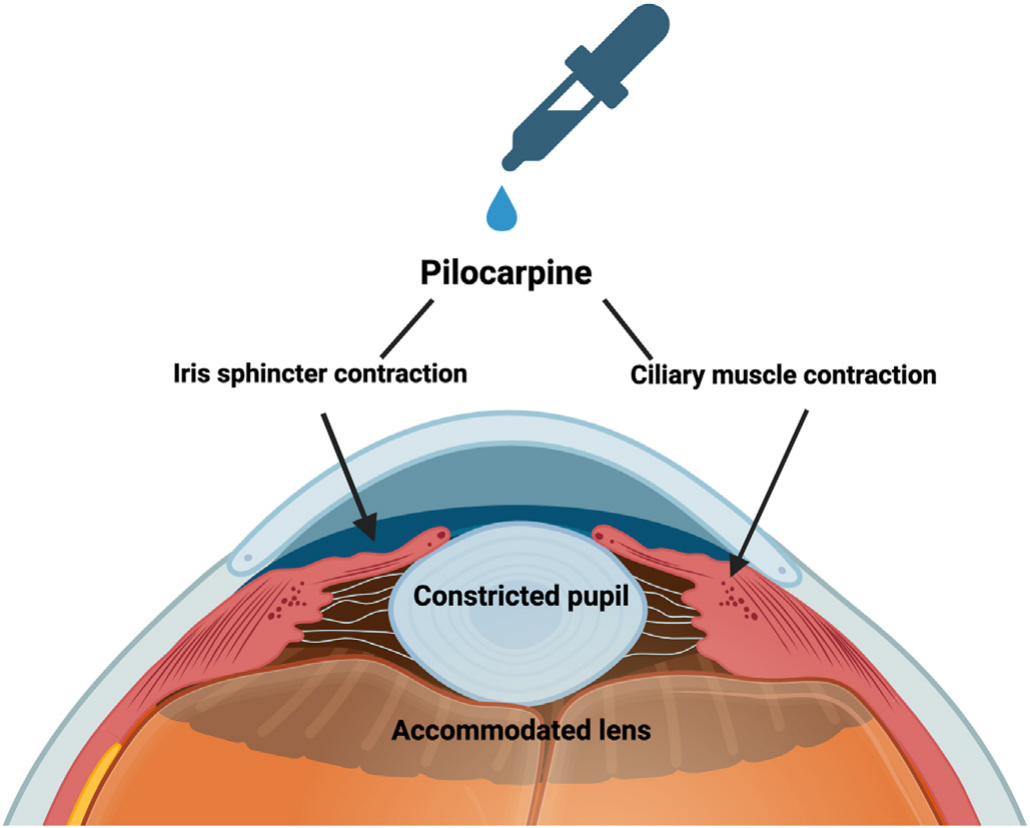
The function chart of Pilocarpine.

Pilocarpine, an alkaloid isolated from the Pilocorpus shrub species, is a direct acting both full and partial cholinergic agonist of the five muscarinic receptor subtypes, especially M3.^26^ Activation of M3 receptors by pilocarpine results in the upregulation of intracellular calcium, leading to contraction of smooth muscles in pupillary sphincter and ciliary bodies.^26^ (Fig. 2).

An optimized topical formulation of pilocarpine HCl 1.25% (Vuity; AGN-190584; Allergan, Inc., an AbbVie company) has become the first pharmacologic treatment of presbyopia approved by the FDA in 2021.^5^ Pooled analysis of two prospective, randomized, vehicle-controlled studies (GEMINI 1 and 2) aimed to assess the effectiveness of topical pilocarpine HCl 1.25% in treating presbyopia while used once daily for 30 days.^27^ Each of the two studies demonstrated notable enhancements in near vision, with subjects achieving an increase of ≥3 lines, while maintaining distance vision with no loss of ≥5 letters. Data from clinical trials also indicated that the solution began to show effect within 15 ​min and lasted for up to 6 ​h.^27^ In the GEMINI studies, the most frequently observed side effects were headaches (14.9%), conjunctival hyperaemia (5.1%), vision blur (4.5%), and eye pain (4.3%).^27^

Another phase 3 randomized, controlled, double-masked, multicentre study (Virgo) investigated the safety, efficacy, and pharmacokinetics of pilocarpine HCl 1.25% administered bilaterally, twice daily (6 ​h apart) for 14 days in participants with presbyopia.^28^ The study met its primary endpoint, which included the proportion of patients gaining ≥3 lines in mesopic, high contrast, binocular distance corrected near visual acuity (DCNVA) with ≤5-letter loss in low light CDVA at Day 14 and hour 9.^28^ Virgo trial results led to FDA approval of this dosing in March 2023. The most frequently observed side effects in the Virgo trial were headaches (8.8%), eye irritation (6.1%), visual impairment (4.4%), punctate keratitis (3.5%), and eye pain (2.6%). There were no reports of retinal or vitreous detachments, retinal tear, or vitreomacular traction, although the end point of the observations was 30 days.^28^ (Table 2).

**Table 2:** Most common treatment-related adverse events of different myotic drugs (TRAE).

Drug	Duration of action	TRAE					
		Conjunctival hyperaemia	Eye irritation	Eye pain	Vision impairment	Headache	Severity of TRAE
Pilocarpine HCl 1.25% (VUITY)	Once daily - 6 ​h	5.1%	–	4.3%	4.5%	14.9%	
	Twice daily - 9 ​h	6.1%	–	2.6%	6.1%	8.8%	82% mild. None severe
Pilocarpine 0.4 (CSF-1)	6-8	1.6%	–	5.8%	3.6%	6.8%	96.1% mild. None severe
Phentolamine ​+ ​Pilocarpine	6	4.55%	6.82%	2.27%	2.27%	0%	None severe
Carbochol ​+ ​Brimonidine (Brimochol)	6–8	–	–	14.04%	–	8.99%	0.56% severe (nephrolithiasis)
Aceclinide ​+ ​Brimonidine (LNZ100)	10	9.0%	20.1%		13.2%	11.5%	Mild

0.4% pilocarpine HCl is the minimum concentration needed to achieve near vision improvement without compromising DVA for presbyopic patients. 0.4% pilocarpine HCl (Qlosi; CSF-1; Orasis Pharmaceuticals) single active ingredient, preservative-free, low-dose eye drop got the FDA approval in 2023 and it was supported by strong efficacy, safety, and tolerability observed in pivotal phase 3 (NEAR 1 and 2) clinical trials.^29^ CSF-1 was administered twice daily for 2 weeks, with efficacy and safety assessments conducted at various intervals. Results showed that CSF-1 met the primary and key secondary endpoints, with a significantly higher percentage of responders achieving ≥3-line gain in NVA compared to the vehicle group. This formulation of low-dose pilocarpine can be dosed up to twice daily, with the second drop installation at around 2–3 ​h after the first drop to extend its duration to 8 ​h.^29^ In the NEAR trials side effects were reported by 16.6% of participants, with 96.1% of these events rated as mild. The most common side effects were headaches (6.8%), instillation site pain (5.8%), blurred vision (3.6%) and conjunctival hyperaemia (1.6%).^29^ (Table 2).

### 4.2 Side effects of pilocarpine

The physiological and pharmacological effects of miotics on the eye suggest that they may pose a risk for retinal tears and detachments. During accommodation the ora serrata and the choroid move forward. The extent of this is about 0.05 ​mm for every diopter of accommodative effort. When pilocarpine is introduced, it intensifies the accommodation response. The lens's posterior surface moves forward significantly during accommodation which not only alters the positioning of the lens but also disturbs the vitreous. Such disturbances in the anterior vitreous can transmit forces to the vitreoretinal interface. If there are abnormal attachments at this interface, the additional movement caused by miotics can exert a pull on these areas. While a normal vitreoretinal attachment might withstand this pull, abnormal attachments are at higher risk for tractional forces that could lead to retinal tears or detachment.^30^

There are few case reports about retinal detachment after installation of pilocarpine solutions. A multicentre case series were reported of 3 eyes from 2 patients with retinal detachments associated with the use of 1.25% pilocarpine drops. The first patient noticed symptoms (flashes and floaters) after 3 days of initiating treatment in both eyes, while the second – after 5 weeks. Both cases involved detachments in different quadrants of the retina with associated tears.^31^

Another two cases were reported of unilateral retinal detachment after 10 days of the initiation of 1.25% pilocarpine drops. Patients were pseudophakic men in their 60 s–70 s with pre-existing retinal detachment risk factors (high myopia, lattice degeneration, and prior retinal detachment). Both affected eyes were treated with pars plana vitrectomy and gas endotamponade with an uncomplicated postoperative course.^32^

Another case was reported of a woman in her 60 s who developed vitreomacular traction immediately following the first administration of pilocarpine 1.25% eye drops.^33^

One more case was reported of transient bilateral vitreomacular traction syndrome after 6 weeks of treatment with 1% pilocarpine ophthalmic solution in both eyes for advanced glaucoma. After the discontinuation of the drug the vitreomacular tractions resolved without a complete posterior vitreous detachment in both eyes.^34^ 1% pilocarpine is investigated in clinical trial for the use in presbyopia treatment also.^17^

There are no cases reported to this day of retinal or vitreous detachments following the use of 0.4% pilocarpine HCl.

Before prescribing pilocarpine it's crucial to evaluate the risk of retinal detachment. Myopic patient's fundus should be examined prior to drops prescription to evaluate the risk factors. Patients should also be educated on the early warning signs of retinal tears or detachment, such as the sudden appearance of flashes, floaters, or loss of visual field before starting the medication.^31^ Pilocarpine solutions are also not recommended to be used with acute iritis because syneachia may form between the iris and the lens.

Furthermore, when pilocarpine is used chronically, it stimulates the uveal tract and can produce adverse effects like inflammatory reactions, pigment dispersion, posterior synechiae, spasmic contractions of the ciliary muscle and iris, which can result in fixed pupil and myopic shift.^35^ To this day there are no long-term complications reported while using pilocarpine for presbyopia treatment.

### 4.3 Other miotic agents

#### 4.3.1 Combinations with pilocarpine

In the previous reviews several combinations of miotic agents with other agents were described.^7^^,^^9^ Combined ocular drop formulations are thought to offer therapeutic advantages for presbyopia treatment, potentially by optimizing miotics activity, gaining additional effect and reducing side effects from miotic agents. Since the last review, there have been no new developments or reported outcomes from mentioned clinical trials.

Combination of pilocarpine (0.247%) and phenylephrine (0.78%) (FOV Tears) for presbyopia treatment was evaluated in case series which included 363 subjects.^36^ Phenylephrine counteracts pupil constriction to avoid posterior synechiae and complications under low-light activities. The study showed a notable 2-line improvement in NVA, without a noticeable change in the photopic pupil size 2 ​h after the administration of FOV Tears. Additionally, the study observed a minimal myopic shift, measured at 0.17 diopters, suggesting the absence of a significant spasm in accommodation.^36^ FOV tears also do not have an impact on the tear film, corneal endothelium, or endothelial cells. Further research is necessary to evaluate the long-term effect of the agent and risks of retinal detachment associated with using a reduced concentration of pilocarpine.

#### 4.3.2 Phentolamine

Phentolamine is a non-selective alpha-adrenergic blocker used primarily for its vasodilatory effects on blood vessels. Phentolamine's mechanism of action involves blocking alpha-1 and alpha-2 receptors, which leads to the relaxation of smooth muscles and thus vasodilation. When used in the eyes, it reduces the pupil diameter, which creates a pinhole effect to improve focus. Due to this mechanism of action which does not engage the ciliary muscle, it could potentially lower the chances of retina traction.^15^

Phentolamine in combination with low dose pilocarpine 0.4% was evaluated in VEGA-1 trial resulting in favourable effect with ≥3 lines of binocular NVA in photopic lighting. This combination also showed good efficacy, safety, and durability. VEGA-2 trial has begun in 2023 to evaluate safety and efficacy of this solution.^15^ The most common side effects are mentioned in Table 2.

0.75% Phentolamine (Nyxol) was approved by the FDA in 2023 for the treatment of pharmacologically induced mydriasis produced by adrenergic agonist or parasympatholytic agents, or a combination of them with the rapid return of dilated pupils to their baseline diameter in 60–90 ​min. Clinical trials also showed an acceptable safety profile of Nyxol for the pediatric population.^37^

#### 4.3.3 Carbachol and brimonidine tartrate

Carbachol is a parasympathomimetic that acts as an agonist of muscarinic and nicotinic receptors. Unlike pilocarpine which only bounds on M3 muscarinic receptors, carbachol is a full agonist promoting great amounts of acetylcholine release from parasympathetic nerve endings. Low carbachol concentration (2.25%) is equivalent to about 3% pilocarpine.^38^

Carbachol is known for its potent, long-lasting miotic effect, leading to pupil constriction.

Brimonidine is an adrenergic agonist with a high affinity for the alpha-2 receptors and it interacts with receptors found on the presynaptic nerve endings associated with the dilator muscle, potentially mitigating the side effects commonly associated with miotics.^39^ Brimonidine also effectively diminish the activity of the dilator muscle. Carbachol combination with brimonidine may extend the therapeutic effect of miotic by affecting aqueous humor dynamics, thus prolonging the improved NVA.^38^

Clinical trial BRIO-I (NCT05270863) evaluated safety and efficacy of the Brimochol (Carbachol 2.75% and Brimonidine 0.1%).^16^ The study met its primary endpoint, prespecified by the FDA, achieving ≥15 ETDRS letter gain in best near visual acuity without a loss of ≥5 letters at distance across all time points through 6 ​h period. The most experienced side effects with Brimochol were eye irritation (14%) and headache (9%). There were no serious adverse events related to any of the treatments in the BRIO-I study, including no cases of retinal detachment, although the follow-up time was very short (Table 2). Phase 3 vehicle-controlled BRIO-II trial is initiated to evaluate 6-month safety and efficacy of Brimochol. It is anticipated to submit the drug to treat presbyopia for the FDA approval in 2024.

It could be anticipated that Brimochol might induce miotic-related side effects like Pilocarpine since its mechanism of action is similar or even stronger than Pilocarpines'. Long follow-up studies are required to evaluate Brimochol's potential side effects.

#### 4.3.4 Aceclidine and brimonidine

Aceclidine is a selective cholinergic miotic agent that binds to acetylcholine receptors in the autonomic nervous system. Aceclidine targets the pupil sphincter muscle with relatively minimal effect on the ciliary muscle.^40^ Compared to nonselective miotics, such as pilocarpine and carbachol, aceclidine's relative stimulation of the ciliary muscle is significantly less and, as such, induces far less myopic shift, lens thickening, and lens anterior displacement. Since it stimulates ciliary muscle less than pilocarpine chances of retina or vitreous tractions should be very small. The combination with Brimonidine causes reduced activity of the dilator muscle producing an even smaller pupil.

In the realm of therapeutic advancements for presbyopia, the company LENZ has been at the forefront with its novel miotics LNZ100 (Aceclidine) and LNZ101 (Aceclidine/Brimonidine), with trials being completed in early 2024.^18^^,^^19^^,^^20^^,^^21^^,^^22^^,^^23^^,^^24^^,^^25^ Characterized by their once-daily application and preservative-free formulation, these drops have exhibited efficacy in enhancing near vision promptly and sustainably, with no adverse effects on distance vision. The phase 2 INSIGHT trial (NCT05294328) assessed the effects of LNZ100 and LNZ101, which include 1.75% aceclidine and brimonidine.^18^ This trial showed encouraging results, indicating that both treatment modalities satisfied the primary efficacy by facilitating ≥ 3-line improvement in VA, without a corresponding loss of ≥1-line in DVA within the first hour post-treatment. LENZ has recently released data from randomized, double-masked, controlled phase 3 CLARITY clinical trials, focusing on the long-term effectiveness and safety of these miotics.^41^ Based on the results of CLARITY 1 and 2 trials (NCT05656027 and NCT05728944), LNZ100 was chosen for the FDA submission in mid-2024 due to its superior performance compared to LNZ101.^19^^,^^22^ LNZ100 showed rapid onset of action with significant improvement in near vision lasting up to 10 ​h. Remarkably, 95% of participants achieved a ≥2-line improvement in near vision after 1 ​h. The treatment was well-tolerated, with most adverse events reported as mild, and no serious treatment-related adverse events were noted in the 42-day follow-up. The common side effects included eye irritation (20.1%), visual impairment (13.2%), and headaches (11.5%) (Table 2). Data from the CLARITY 3 trial, which is expected to provide information on the safety of LNZ100 over a duration of 180 days, is awaited.^41^ More comprehensive results and data analysis are expected to be presented at upcoming industry conferences.

## 5 Conclusions

Undoubtedly, the pharmacological approach to treat presbyopia is emerging as a significant area of interest in ophthalmologic research. FDA approval of Pilocarpine ophthalmic solutions to treat presbyopia was a big milestone in the field and more miotic agents are undergoing promising clinical trials to be approved by the FDA for presbyopia treatment. Despite that, there is still lacking long term follow-up results to evaluate miotic agents’ safety and possible side effects.

## Study approval

Not applicable.

## Author contributions

The authors confirm contribution to the paper as follows: Conception and design of study: AG, LK. RZ; Data collection: LK; Analysis and interpretation of results: LK, AG; Drafting the manuscript: LK, AG, RZ; All authors reviewed the results and approved the final version of the manuscript.

## Funding

This research did not receive any specific grant from funding agencies in the public, commercial, or not-for-profit sectors.

## Declaration of competing interest

The authors declare that they have no known competing financial interests or personal relationships that could have appeared to influence the work reported in this paper.

## Abbreviations

FDA U.S.: Food and Drug Administration
NVA: near visual acuity
DVA: distance visual acuity
BCDVA: best distance corrected visual acuity
CNVA: corrected near visual acuity
LNZ101: Aceclidine/Brimonidine
LNZ100: Aceclidine
